# Use of flexible sensor to characterize biomechanics of canine skin

**DOI:** 10.1186/s12917-018-1755-y

**Published:** 2019-01-25

**Authors:** Austin R. J. Downey, Jin Yan, Eric M. Zellner, Karl H. Kraus, Iris V. Rivero, Simon Laflamme

**Affiliations:** 1 Department of Mechanical Engineering, University of South Carolina, Columbia, South Carolina United States; 2 Department of Civil, Construction, and Environmental Engineering, Iowa State University, Ames, Iowa United States; 3 Department of Veterinary Clinical Sciences, Iowa State University, 1809 S Riverside Dr, Ames, 50011-3619 Iowa United States; 4 Department of Industrial and Systems Engineering, Rochester Institute of Technology, Rochester, New York United States; 5 Department of Electrical and Computer Engineering, Iowa State University, Ames, Iowa United States

**Keywords:** Soft elastomeric capacitor, Biomechanics, Canine skin, Strain measurement, Biomedical measurement, Polymers

## Abstract

**Background:**

Suture materials and techniques are frequently evaluated in ex vivo studies by comparing tensile strengths. However, the direct measurement techniques to obtain the tensile forces in canine skin are not available, and, therefore, the conditions suture lines undergo is unknown. A soft elastomeric capacitor is used to monitor deformation in the skin over time by sensing strain. This sensor was applied to a sample of canine skin to evaluate its capacity to sense strain in the sample while loaded in a dynamic material testing machine. The measured strain of the sensor was compared with the strain measured by the dynamic testing machine. The sample of skin was evaluated with and without the sensor adhered.

**Results:**

In this study, the soft elastomeric capacitor was able to measure strain and a correlation was made to stress using a modified Kelvin-Voigt model for the canine skin sample. The sensor significantly increases the stiffness of canine skin when applied which required the derivation of mechanical models for interpretation of the results.

**Conclusions:**

Flexible sensors can be applied to canine skin to investigate the inherent biomechanical properties. These sensors need to be lightweight and highly elastic to avoid interference with the stress across a suture line. The sensor studied here serves as a prototype for future sensor development and has demonstrated that a lightweight highly elastic sensor is needed to decrease the effect on the sensor/skin construct. Further studies are required for biomechanical characterization of canine skin.

**Electronic supplementary material:**

The online version of this article (10.1186/s12917-018-1755-y) contains supplementary material, which is available to authorized users.

## Background

In veterinary medicine, incisional dehiscence is a known complication of wound closures under tension or in areas of high motion. Axial pattern flaps are one example of these wound closures which experience at least a partial dehiscence in 20–30% of cases [1–3]. Routine veterinary surgical procedures have dehiscence rates of 2–3% [4]. In humans, there is a wide range of dehiscence rates from 2% of all orthopedic surgeries [5] to 9–10% of leg wound closures [6].

Many surgical devices and techniques are used for the closure and repair of skin defects in veterinary and human medicine. These various methods are often compared to each other in experimental models or laboratory settings to investigate the optimal surgical technique for wound closure [7–11]. However, the actual loads and displacements of those tissues are unknown in most species, even humans, making experimental performance of limited value to clinical practice. With a better understanding of the biomechanics of skin wounds, common postoperative complications such as dehiscence may be avoided or additional therapies implemented prior to occurrence.

One method for monitoring skin deformation (i.e. strain) in vivo is digital image correlation [12, 13]. While effective in obtaining full-field strain maps, this technique is not well suited for the continuous monitoring of patients during recovery, or monitoring of suture lines under bandages. The use of flexible electronics for the measuring of biomechanical movement has seen a high level of research interests in recent years [14–17]. These studies focus on the development of the technology and less on the application of the technology in the medical field. In this work, a novel large area electronic that has been studied for use in the monitoring of civil infrastructure [18–20] is investigated for use in monitoring the high levels of strain present on the surface of skin. This sensor, termed soft elastomeric capacitor (SEC), is a large area electronic that is highly flexible, elastic and easily customizable in both shape and size. The future goal of this application is to understand the actual tensile forces across skin at rest and during activity. An altered Kelvin–Voigt material model is utilized to map the SEC’s measured strain to an estimated stress in a skin sample under the sensor. In this paper, we report the findings of an ex vivo study on the information obtained from a sensor adhered onto canine skin. We hypothesize that the sensor would record strain proportional to that introduced into the skin by the material testing machine used.

## Soft elastomeric capacitor

The SEC is a robust, elastic, inexpensive large area electronic that is easy to fabricate and customizable in shape and size. The SEC is a parallel plate capacitor where the capacitor’s (sensor’s) dielectric is composed of a styrene-ethylene-butylene-styrene (SEBS) block co-polymer matrix filled with titania (TiO_2_). The titania is added to increase both its durability and permittivity. A dielectric mix is fabricated through the mixing and sonication of styrene-ethylene-butylene-styrene (SEBS) and titania into toluene. This solution is drop cast onto a flat glass plate to produce a dielectric. Once dry, two conductive plates are painted onto each side of the dielectric using a conductive paint fabricated from the same SEBS matrix, but filled with carbon black particles. Lastly, copper contacts with a conductive adhesive are added to the sensor to allow for the signal wire, and therefore data acquisition (DAQ) systems, to be connected through the use of a soldered connection. For more details on the sensor’s fabrication procedure, the interested reader is referred to reference [18].

**Fig. 1:**
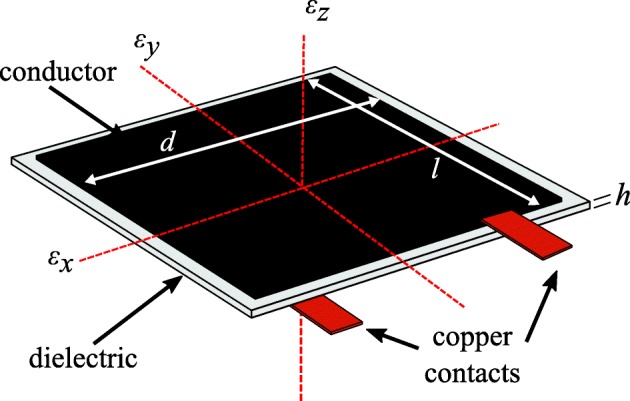
Soft elastomeric capacitor (SEC) sensor with axes and key components annotated

The SEC transduces a change in its geometry (i.e., strain), into a measurable change in capacitance. Assuming a sampling rate of less than 1 kHz, the SEC can be modeled as a non-lossy capacitor by

1 $$ C=e_{0}e_{r} \frac{A}{h}   $$

where *C* is the capacitance of the sensor, *e*_0_=8.854 pF/m is the vacuum permittivity, *e*_*r*_ is the polymer relative permittivity, *A*=*d*·*l* is the sensor area of width *d* and length *l*, and *h* is the thickness of the dielectric as annotated in Fig. 1.

### Experimental testing

**Fig. 2:**
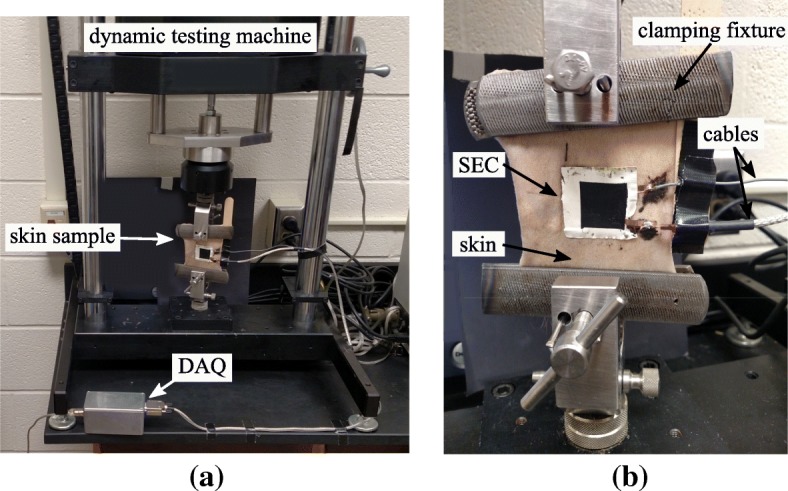
Experimental test configuration of the canine skin with the SEC showing the: **a** canine skin with an attached SEC sensor in the dynamic testing machine; and **b** a close up of the SEC sensor adhered onto the canine skin

The experimental configuration was designed to investigate the capability of the SEC to monitor the forces present in the canine skin. A 1–2 year old, euhydrated frozen (-20 C) beagle skin sample from the dorso-lateral thorax, euthanized for reasons unrelated to this study, was allowed to thaw overnight at room temperature before being utilized in this study. While the skin sample was tested at room temperature and no cold spots were felt in the sample, the effects of the freeze-thaw cycle on the skin’s mechanical properties are unknown. Readers are cautioned in utilizing our measurements to represent in vivo biomechanics of skin due to the limitations of the current study, specifically the ex vivo nature of the experiment, sample preservation prior to investigation, and the altered skin response due to the SEC. Previous studies noted that freezing skin at -20 C preserves the normal elasticity of the specimen [21]. However, it is also noted that any method of conservation of samples will change the biomechanics of the tissues [22–24]. In most cases, the behavior of a preserved specimen will be similar yet increased or decreased from the normal mechanical behavior of fresh samples. An SEC sensor was attached to the canine skin sample and placed in a dynamic testing machine as shown in Fig. 2a. The canine skin, not including the skin material under the connections, measured approximately 76 × 87 × 2 mm^3^. The SEC was adhered onto the canine skin using a commercial two-part epoxy (JB-Weld). The epoxy applied under the top and bottom contacts to prevent the contacts from moving during testing (Additional file 2). This two-part epoxy adds a lateral stiffness to the sensors after curing, helping to ensure that the sensor extends and deforms in the same manner as the sensor used for calibration. The canine skin was gripped between two barbed clamping fixtures (hand rasps cut in half) and mounted onto the dynamic testing machine. Once the skin was mounted in the dynamic testing machine, a support was added to the wires to ensure that their self-weight did not interfere with the measurements, as denoted in Fig. 2b. In addition to testing the canine skin with the SEC, (Additional file 1) the same canine skin sample was also tested without the SEC to obtain its material properties. In this case, the canine skin was mounted similarly to that shown in Fig. 2, however, without the SEC skin attached.

The skin was mechanically excited with a displacement controlled 0.1 Hz harmonic load with 4.1 mm amplitude. As before, the gauge length of the SEC was set to 10 mm. The skin was not pretensioned in the dynamic testing machine before testing. This allowed the skin to go to slack during the lower portions of the displacement loading and also allowed the introduction of an out-of-plane deformation into the skin. To account for this out-of-plane deformation, only the part of the loading cycle where the skin is fully tensioned is considered during modeling, presented later in this work. A custom made DAQ was attached to the SEC. This DAQ consists of a capacitance measurement device and shield driver for eliminating the parasitic capacitance found in the signal wire. SEC capacitance data (Additional file 3) was sampled at 20 samples per second (S/s) and recorded on a laptop.

### Mechanical system modeling

**Fig. 3:**
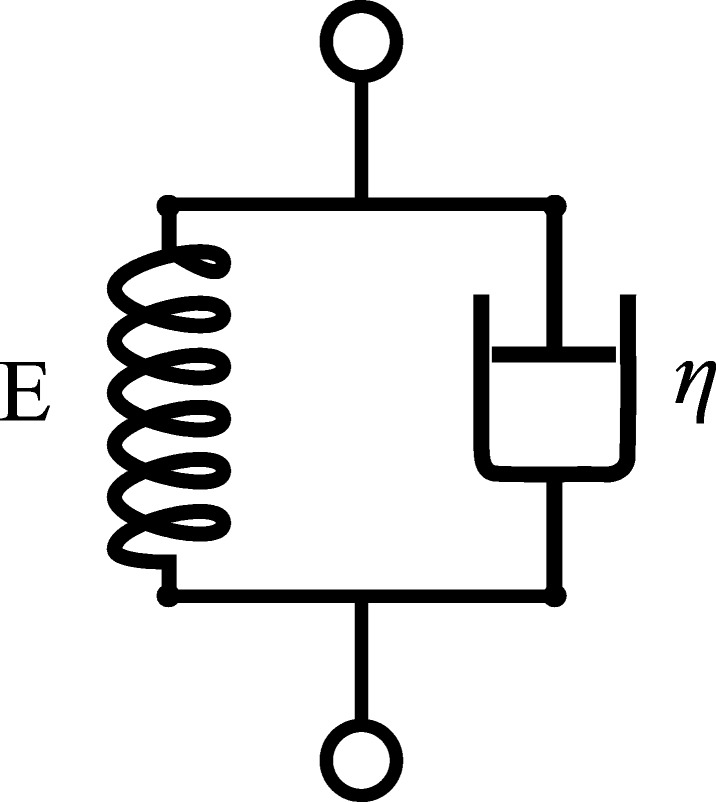
Kelvin-Voigt model for a viscoelastic material where E is a modulus of elasticity and *η* is the viscosity

To map the measured strain in the SEC (converted from the SEC’s capacitance using Eq. 14) to forces in the canine skin, a modified Kelvin-Voigt material is adopted where the stress for a material is defined as a typical Kelvin-Voigt material:

2 $$ \sigma(t) = \mathrm{E} \varepsilon({t}) + \eta \frac{\mathrm{d} \varepsilon({t})}{\text{dt}}   $$

where *σ*(*t*) is the stress in the material, as a function of time, and *E* and *η* are the materials modulus of elasticity and viscosity, respectively. Equation 2 is diagrammed in Fig. 3 as a spring and dashpot in parallel. This work assumes that stress values calculated here are constant under the SEC sensor, a modeling approach considered in other ex-vivo material properties of skin [25]. To account for nonlinearities in the canine skin, E and *η* are considered to be functions of the current level of strain (*ε*) in the material. Here, we defined E and *η* such that

3 $$ \mathrm{E} = a+b \cdot e^{c \varepsilon} \quad \text{and} \quad \eta = x+y \cdot e^{z \varepsilon}  $$

therefore, Eq. 2 can be expressed as

4 $$ \sigma(t) = \left(a+b \cdot e^{c \varepsilon}\right)\varepsilon(t) + \left(x+y \cdot e^{z \varepsilon}\right)\frac{d \varepsilon(t)}{dt}  $$

where *a*, *b*, *c*, *x*, *y*, and *z*, are solved for using a particle swarm algorithm [26]. These parameters are solved for both the canine skin without the SEC sensor and the canine skin with the SEC sensor. Once solved, the strain-dependent Kelvin-Voigt material models are defined by the parameters E _skin-data_, *η*_*skin*−*data*_, E _skin-SEC-data_, and *η*_*skin*−*SEC*−*data*_ where the subscript indicates that they are the values associated with the data set for either the canine skin or the canine skin with an SEC attached.

**Fig. 4:**
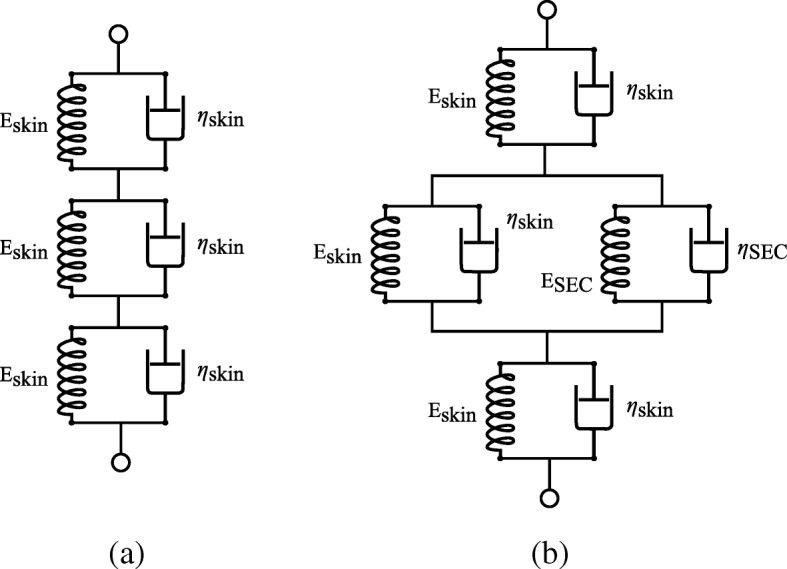
Mechanical models used for the: **a** canine skin without the SEC sensor; and **b** and the canine skin with the SEC sensor

Spring and dashpot representations for the “canine skin system”, and “canine skin and SEC system” can be constructed using a system of Kelvin-Voigt material models as shown in Fig. 4. Here, Fig. 4a is a representation of the canine skin and Fig. 4b is a representation of the canine skin with the SEC added in parallel to only the center portion of the skin. From the Kelvin-Voigt material models in Fig. 4a, the material stiffnesses can be added such that

5 $$ \text{E}_{\text{skin-data}} = \frac{1}{\frac{1}{\text{E}_{\text{skin}}}+\frac{1}{\text{E}_{\text{skin}}}+\frac{1}{\text{E}_{\text{skin}}}}  $$

or:

6 $$ \text{E}_{\text{skin}} = 3 \cdot \text{E}_{\text{skin-data}}   $$

and the material viscosity parameters are combined such that:

7 $$ \mathrm{\eta}_{\text{skin-data}} = \frac{1}{\frac{1}{\mathrm{\eta}_{\text{skin}}}+\frac{1}{\mathrm{\eta}_{\text{skin}}}+\frac{1}{\mathrm{\eta}_{\text{skin}}}}  $$

or:

8 $$ \mathrm{\eta}_{\text{skin}} = 3 \cdot \mathrm{\eta}_{\mathrm{skin-data}}   $$

A similar approach can be taken with the models in Fig. 4b. Given that the SEC sensor and the canine skin in the center section will experience the same level of strain (*ε*_SEC_=*ε*_skin_), we can define E_skin-SEC-data_ as:

9 $$ \mathrm{E}_{\text{skin-SEC-data}} = \frac{1}{\frac{1}{\mathrm{E}_{\text{skin}}}+\frac{1}{\mathrm{E}_{\text{skin}}+\mathrm{E}_{\text{SEC}}}+\frac{1}{\mathrm{E}_{\text{skin}}}}  $$

therefore:

10 $$ \mathrm{E}_{\text{SEC}} = \frac{-(3 \cdot \mathrm{E}_{\mathrm{skin-SEC-data}} - \mathrm{E}_{\text{skin}}) \mathrm{E}_{\text{skin}}}{2 \cdot \mathrm{E}_{\text{skin-SEC-data}} - \mathrm{E}_{\text{skin}}}   $$

We can solve for *η*_*SEC*_, again taking *ε*_SEC_=*ε*_skin_ for the center section:

11 $$ \eta_{\text{skin-SEC-data}} = \frac{1}{\frac{1}{\mathrm{\eta}_{\text{SEC}}} + \frac{1}{\mathrm{\eta}_{\text{skin}}+\mathrm{\eta}_{\text{SEC}}} + \frac{1}{\mathrm{\eta}_{\text{skin}}} }  $$

therefore:

12 $$ \mathrm{\eta}_{\text{SEC}} = \frac{-(3 \cdot \mathrm{\eta}_{\text{skin-SEC-data}} - \mathrm{\eta}_{\text{skin}}) \mathrm{\eta}_{\text{skin}} }{2 \cdot \mathrm{\eta}_{\text{skin-SEC-data}} - \mathrm{\eta E}_{\text{skin}}}   $$

Equations 6, 8, 10, and 12 can be used with Eq. 2 to solve for the stress in either the SEC or a subset of the canine skin. Or these parameters can be combined to solve for the total stress in the system. Knowing that the stress (*σ*) must be equal at each section of the model, it can be noted that the total stress in the skin is equal to the summation of the stress in the SEC (*σ*_SEC_) and the stress in the skin directly under the SEC (*σ*_skin_). Therefore, the stress in the section of interest is defined as:

13 $$ \sigma_{\text{skin-SEC}}=\sigma_{\text{SEC}} + \sigma_{\text{skin}}   $$

Once all the material properties have been solved for, the stress in the canine skin under the SEC can be computed using Eq. 2 and the strain data measured through the SEC.

## Results

**Fig. 5:**
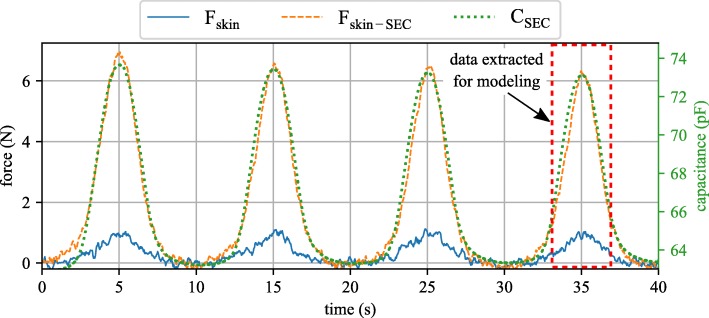
Force and capacitance results for the canine skin (force only) and the canine skin with the SEC sensor attached (force and capacitance)

Figure 5 reports the time series test data captured during testing. The SEC is shown to track the strain in the canine skin, here reported in terms of the force measured by the dynamic testing machine. The flat portion between each peak is due to the out-of-plane deformation (i.e. slack) in the canine skin that is present during this portion of the loading cycle. The SEC being attached to the canine skin can deform with the skin and is, therefore, able to capture the out-of-plane deformation of the canine skin. This deformation manifests itself as a measurable force in the dynamic testing machine, a force that the SEC is shown capable of tracking. A notable stress relaxation can be seen in the stress data of the canine skin with the SEC attached. This stress relaxation does not appear to be present in the canine skin stress data and could potentially be attributed to the SEC. This stress relaxation is a well-documented property in polymers [27, 28] and its effect can be removed if the material is excited for a sufficient number of cycles [29]. For this reason, and the before mentioned out-of-plane deformation present during the lower portion of the cyclic loading, only the data inside the dashed-red box in Fig. 5 was extracted for post-processing. The addition of the SEC to the canine skin was found to add considerable stiffness to the system. This can be quantified by looking at Fig. 5 where the force required to displace the canine skin and SEC sensor is six times that required to displace only the canine skin.

**Fig. 6:**
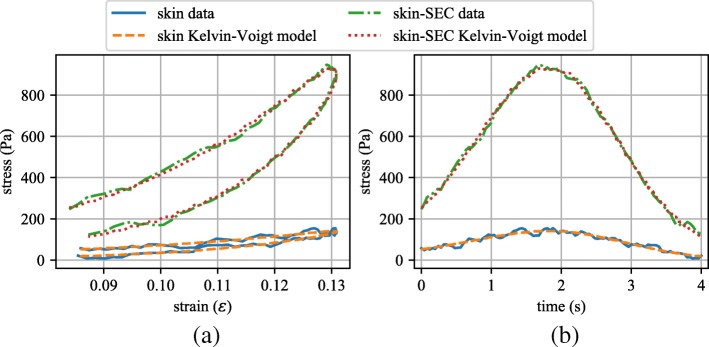
Kelvin–Voigt model fitting results for both the canine skin and the canine skin with the SEC sensor attached expressed in term of: **a** stress and strain; and **b** stress and time

Results for the modified Kelvin-Voigt model as presented in Eq. 4, using the parameters listed in Table 1, are presented in Fig. 6. Figure 6a reports the experimental data and model results for both systems, the canine skin and the canine skin and SEC, in terms of stress and strain while Fig. 6b reports the same data, but in terms of stress and time. The model results are shown to fit the experimental data well.

**Table 1:** Parameters used for the nonlinear Kelvin-Voigt model, as expressed in Eq. 4

	Parameter					
	*a*	*b*	*c*	*x*	*y*	*z*
Canine skin	128	28	27	0	8301	9
Canine skin & SEC sensor	410	144	19	5900	3194	31

**Fig. 7:**
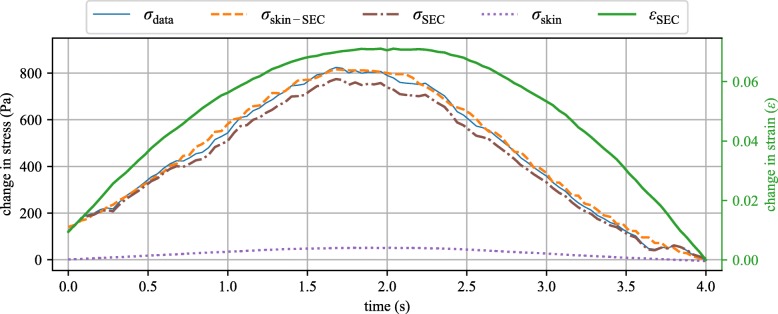
Results showing the stress calculated using the measured load from the dynamic testing machine, the stresses calculated using the Kelvin–Voigt model and the strain measured with the SEC

Lastly, using the model data presented in Fig. 6 along with Eq. 13, the change in stress in the canine skin under the SEC (*σ*_skin_) can be estimated. The stress is in relative terms (i.e., change in stress) because it is calculated from the SEC’s measured strain that was zeroed. The zeroing of the SEC capacitance, and by extension strain, signal is necessary when applying the gauge factor of 0.61. As reported in Fig. 7, the majority of the stress in the canine skin and SEC sensor system is taken by the SEC with only a small amount of stress being taken by the canine skin. This is to be expected as the SEC was shown to add considerable stiffness to the system and is, therefore, much stiffer than the canine skin. In addition to reporting the stress in the canine skin, Fig. 7 reports stress present in the SEC sensors and the stress in the “canine skin and SEC sensor system”. The “canine skin and SEC sensor system” stress is presented for both the experimental data (*σ*_data_) and model (*σ*_skin-SEC_), exhibiting a high level of agreement.

## Discussion

In this study, we presented the feasibility of estimating strain and stress in a canine skin sample with a highly elastic sensor termed soft elastomeric capacitor (SEC) sensor. This non-destructive method requires the use of models to obtain parameters about the skin because the sensor alters the response of the skin due to the direct-contact nature of the method. This method could be useful in tracking biomechanical properties that are unknown for most soft tissues in most species. While the modulus of elasticity is likely variable between species and even breeds, the integration of nondestructive methods to compute a range of possible values would enable further characterizations of such biomechanical properties for canine skin. Monitoring soft tissues over time and during various activities would allow a more evidence-based recommendation to activity restrictions, suture patterns, and tension-relieving techniques.

Our approach is achieved through testing of a canine skin sample both with and without an SEC sensor attached. Once the canine skin has been characterized, the strain in the skin is measured through the SEC sensor. Next, an estimated stress value can be obtained through the assumption of a modified Kelvin-Voigt model, whose material parameters were obtained in prior testing. These calculations assume that a simple Kelvin-Voigt model is sufficient to capture the complex biomechanics of canine skin, assuming the skin does not undergo any out-of-plane deformations during modeling and that the attachment of the SEC to the canine skin does not affect the gauge factor of the SEC. This novel approach to measuring the strain experienced in the skin may be applied with further refinement to a live patient. Variables such as age, breed, and hydration that have been noted to affect the elasticity of skin will pose difficulty in exactly mapping the biomechanics of skin [30]. However, a range of reference values would be useful for designing future surgical implants and performance of ex vivo testing. This information would quantify and further validate Karl Langer’s long accepted skin tension lines map in humans and those adapted for veterinary patients [31, 32]. However, limitations to this proposed method include the measurement and monitoring of out-of-plane motion, challenges with adhering the sensor to a live patient and the interference between bandages and the sensor. Further research is needed on the effects of various adhesives to the skin to determine optimal bonding with minimal effect on stiffening of the skin.

Due to the ex vivo nature of the study, readers are cautioned to utilizing our measurements to represent in vivo biomechanics of skin. Specifically, the sample preservation prior to investigation always affects the mechanical behavior of skin samples. Previous studies noted that freezing skin at -20 C preserves the normal elasticity of the specimen [21]. However, it is also noted that any method of conservation of samples will change the biomechanics of the tissues [22–24]. In most cases, the behavior of a preserved specimen will be similar, yet exaggerated or dampened from the normal mechanical behavior of fresh samples.

With the assumption of the appropriate biomechanic models, these strain measurements can then be related to the stress present in the tissue. With stress representing force per area, this study provides evidence that a sensor may be capable of monitoring stress in skin in vivo. Knowing the actual force in live canine skin under normal conditions is necessary for proper interpretation of the ex vivo studies evaluating suture materials and patterns in skin. At this moment, ex vivo study results are limited to comparing sample groups only to each other instead of in vivo data [7–11]. If canine skin can be biomechanically characterized, reference values of tensile forces may be utilized in evaluation of currently used suture materials and techniques in laboratory settings.

## Conclusion

This study documents the use of an SEC sensor for measurement of strain in canine skin. The values in this study are not intended to be utilized as actual values of canine skin as the sample was not fresh and the effects of the freeze-thaw cycle on skin is unknown. Further investigation of flexible sensors is needed for characterization of canine skin on the live patient.

## Methodology

This section introduces the methodology used for estimating the forces present in a canine skin sample. First, the calibration procedure for the SEC sensor is described. Second, the experimental setup involving the canine skin is introduced. Lastly, the mechanical models used for estimating the forces in the canine skin are presented.

### Sensor calibration

**Fig. 8:**
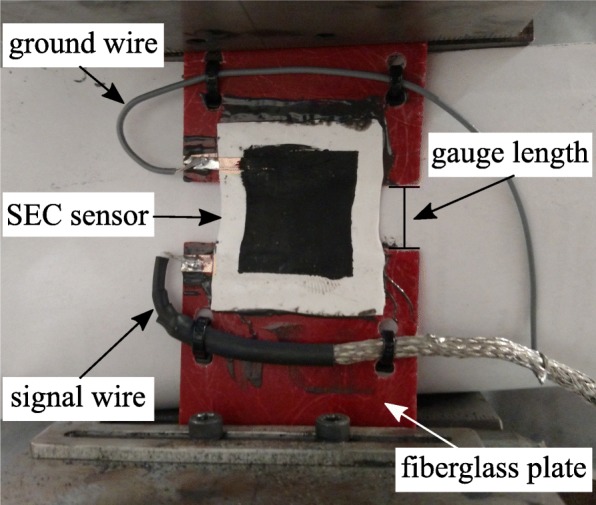
Test configuration used for calibrating the SEC sensor

**Fig. 9:**
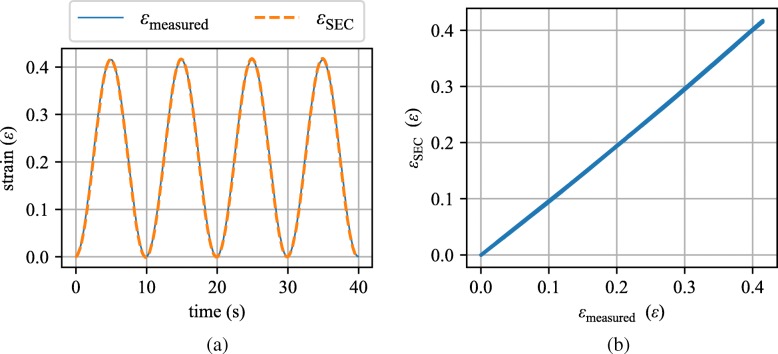
Computed input strain and SEC-estimated strain data for the calibration test presented: **a** as a time series; and **b** as *ε*_SEC_/*ε*_measured_

A free-standing calibration of the SEC was performed through attaching an SEC to a fiberglass substrate (1.58 mm thick) as shown in Fig. 8. The SEC was adhered onto the substrate using a commercial off the shelf epoxy (JB-Weld). As this study is primarily focused on estimation of the stress in skin measured with an SEC, the proper adhesive (likely a skin acrylate) will need to be investigated in future research. To help ensure a constant connection between the copper contacts and the conductive plate of the SEC, the contacts were also adhered onto the fiberglass substrate. The gauge length, defined as the distance between the fiberglass plates, was set to 10 mm. The SEC sensor was mechanically excited by a harmonic load of 0.1 Hz with a 4.1 mm amplitude. The sensors’ unidirectional strain (*ε*), assuming plain stress and no out-of-plane deformations [19], for the freestanding configuration can be

14 $$ \frac{\Delta C}{C}=\lambda \varepsilon   $$

where *λ* represents the gauge factor of the sensor and *Δ*C denotes a change in capacitance. Here, the gauge factor was found experimentally to be 0.61 for the free-standing configuration shown in Fig. 8. The time series strain values computed from the dynamic testing machine measurements (*ε*_measured_) and the strain measured by the SEC (*ε*_SEC_) using Eq. 14 are presented in Fig. 9a. Additionally, the linearity between the strain measured by the dynamic testing machine (applied) and that measured by the SEC are shown in Fig. 9b. Results demonstrate the SEC, using a gauge factor of 0.61, can be used to measure high levels of strain (here 40%) repeatably.

## Additional files

Additional file 1 Load frame data for test without SEC attached to sample. The raw, unfiltered, experimental data collected from the load frame during testing. This data includes time, applied displacement, and measured force. (TXT 67 kb)

Additional file 2 Load frame data for test with SEC attached to sample. The raw, unfiltered, experimental data collected from the load frame during testing. This data includes time, applied displacement, and measured force. (TXT 67 kb)

Additional file 3 Capacitance data from the SEC for the experiment test. The raw, unfiltered, experimental data collected from the SEC DAQ. (TXT 152 kb)

## Abbreviations

DAQ: Data acquisition
SEBS: Styrene-ethylene-butylene-styrene
SEC: Soft elastomeric capacitor
